# Regarding “Loss of seton in patients with complex anal fistula: a retrospective comparison of conventional knotted loose seton and knot‑free seton” by Verkade et al

**DOI:** 10.1007/s10151-022-02637-6

**Published:** 2022-06-20

**Authors:** T. Horeman-Franse

**Affiliations:** Delft University of Technology, Delft, The Netherlands

Dear Sir,

I am the original inventor and principal investigator of the SuperSeton (Medishield B.V., Delft, The Netherlands) and with great interest I have read the article by Verkade et al. in which a group of patients treated with knotted setons (*n* = 67) was compared with a group treated with knot-free setons (*n* = 217) [1]. The authors concluded that loss of seton (LOS) occurs frequently in patients treated for complex anal fistulas and that the incidence of LOS is significantly higher in patients treated with a knot-free loose seton. It was particularly interesting to read that the authors used two different types of knot-less setons in the knot-free seton group, the original “SuperSeton” [2–4] and a clone named “Comfort Drain” (A.M.I., Feldkirch, Austria). As a scientist, I found that combining these two different implants into one category is not scientifically correct; moreover, the retailer that provided the SuperSeton to the researchers informed me that among the 217 knot-free setons provided to the authors there were only ten SuperSetons.

Figure 1A shows that the original SuperSeton design makes use of two wedges while the clone only has a somewhat wedged tip with poorly defined rim. It also shows that the moulding process of the polypropylene materials results in an accurately defined shape with sharp wedges in case of the SuperSeton insert. In addition, the SuperSeton comes with an applier that allows the user to establish the connection without bending the insert or compromising its structural integrity [1]. Figure 1D shows how this applier allows linear expansion of the SuperSeton tube during insertion of the Seton insert. Without this applier, a reliable connection cannot be made which is possibly the reason why the authors concluded “Further developments in seton manufacturing should be focussed on optimisation of the closure mechanism”. Figure 1B shows a tensile strength setup that was used to measure and compare the original design and clone. The results indicated that a mean difference of > 40% in maximum force was found between the two different seton designs before the connection failed.

**Fig. 1:**
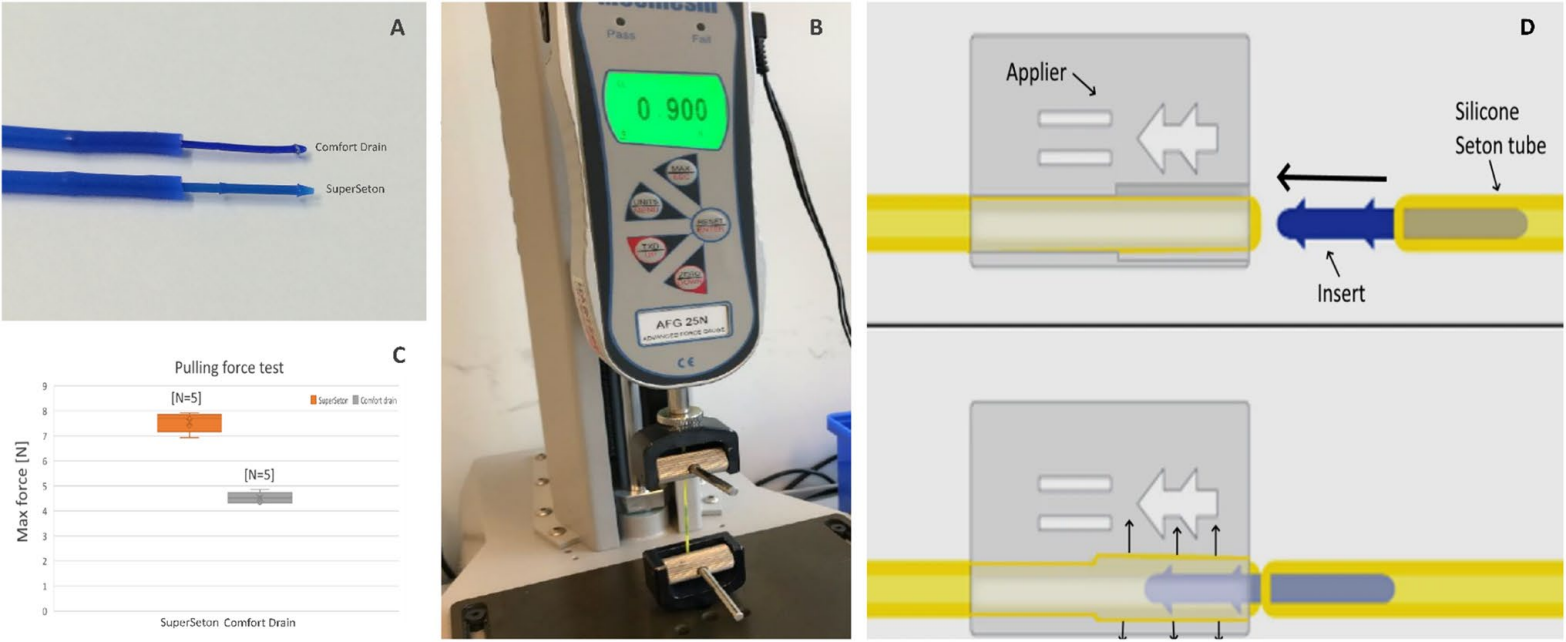
Comparison between the original SuperSeton and Comfort Drain clone. **A** The original Superseton has sharp edges and two arrow shaped wedges. The clone has a tempered tip only. **B** Setup used to measure the maximum force before dislodging. **C** Measurements show that the two seton designs have different dislodgement forces. **D** The applier allows the tube to expand in a controlled way during insertion of the insert. This is essential for a reliable connection

By analysing the LOS results for the knot-free seton patients as a single group without deviating between the two different designs, two things happen. First, a very large deviation was found with a LOS of 89 days (range 5–1039 days) in the study of Verkade et al. as the clones will hypothetically dislodge in the first months while the original SuperSeton will stay in place much longer. Second, the results do not correlate with a previous study in Nature Scientific by Stellingwerf et al. [2] that shows a LOS of 34.8% for knotted setons versus 12.7% for the SuperSeton after 3 months. Therefore, concluding that the SuperSeton is inferior to the knotted seton is invalid and misleading to the readership as the data only show that a group of patients treated with the inferior Comfort Drain mixed with some original SuperSetons had a higher LOS compared to a patient group treated with a knotted seton, without specifying how much of each were used. Therefore, I encourage the authors of the article to make the data accessible to the readership and to split the knot-less patient group into two groups, one group treated with the original SuperSeton and one group treated with the Comfort Drain. By splitting the single knot-free three group in two independent groups (10 SuperSetons and 207 Comfort Drains) and by explaining whether the connection was established with the applier according to the instructions for use. A fair comparison can then be made and the relevance and scientific integrity of this interesting article will be restored.

## Declarations

### Reserach involving human particpiants and/or animals

This article does not contain any studies with human participants or animals performed by any of the authors.
